# Klotho-related Molecules Upregulated by Smoking Habit in Apparently Healthy Men: A Cross-sectional Study

**DOI:** 10.1038/srep14230

**Published:** 2015-09-18

**Authors:** Kaori Nakanishi, Makoto Nishida, Masaya Harada, Tohru Ohama, Noritaka Kawada, Masaaki Murakami, Toshiki Moriyama, Keiko Yamauchi-Takihara

**Affiliations:** 1 Health Care Center, Osaka University, Toyonaka, Osaka, Japan; 2 Laboratory of Developmental Immunology, JST-CREST, Graduate School of Frontier Biosciences, Graduate School of Medicine, and WPI Immunology Frontier Research Center, Osaka University, Suita, Osaka, Japan

## Abstract

While aging is unavoidable, the aging mechanism is still unclear because of its complexity. Smoking causes premature death and is considered as an environmental aging accelerator. In the present study, we focused on the influence of smoking to the serum concentration of anti-aging protein α-klotho (αKl) and the β-klotho-associated protein fibroblast growth factor (FGF)-21 in men. Subjects consisted of apparently healthy men over 40 years of age who underwent health examination. Physical and biochemical parameters, including the levels of several cytokines and growth factors, were obtained from the subjects. Among middle-aged men (46.1 ± 5.1 years), serum levels of FGF-21, soluble αKl (sαKl), and inflammation-related cytokine interleukin (IL)-6 were significantly higher in smokers than in never-smokers. Serum levels of FGF-21 increased and correlated with alanine transaminase, γ guanosine-5′-triphosphate, and total cholesterol only in smokers, suggesting FGF-21 as a metabolic disorder-related factor in smokers. In aged men (60.3 ± 1.7 years), although the serum levels of sαKl in never-smokers were low, smokers showed highly increased serum levels of sαKl. Serum levels of sαKl was correlated with IL-6 in middle-aged never-smokers, suggesting sαKl regulates IL-6. However, this correlation was disrupted in smokers and aged men.

Smoking is one of the major public health problems in modern society. According to data from the World Health Organization (WHO), more than 1.1 billion people worldwide are smokers and half of them die from smoking-related diseases. It is also estimated that by the year 2030, more than eight million people per year will die due to smoking^1^. The leading causes of death by smoking are cancer, cardiovascular disease, and pulmonary disease, which are also known as aging-related diseases^2^,^3^. Smoking also promotes other classical aging-related diseases, such as osteoporosis and macular degeneration and is considered as an environmental aging accelerator^3^. In addition, smoking causes premature death, and the life-span of smokers is more than 10 years shorter than that of never-smokers^2^. Although the acceleration of aging induced by smoking was reported in many previous studies, its precise mechanism is still unclear. Aging is a complicated phenomenon and is influenced by various genetic and environmental factors^4^.

In the present study, we focused on the anti-aging molecule α-klotho (αKl). α*Kl* is a well-established anti-aging gene and the gene mutant mice have been shown to have short life-spans and multiple aging phenotypes analogous to those observed in humans, such as skin atrophy, osteoporosis, ectopic calcification, atherosclerosis, and pulmonary emphysema^5^,^6^. The αKl gene encodes a single-pass transmembrane protein and is primarily expressed in the distal tubules of kidney, parathyroid gland, and choroid plexus. It also exists in soluble form, which is produced by shedding the transmembrane form^7^. Membrane form αKl functions as a co-receptor for fibroblast growth factor (FGF)-23, while soluble form αKl is a kind of enzyme^8^. It has been suggested that both forms play an essential role in mineral-ion homeostasis and loss of this homeostatic function might have shorten the life-spans of the gene mutant mice^9^.

A homolog of αKl, named β-klotho (βKl), interacts with FGF-19, which is known as a regulator of bile acid homeostasis^10^. βKl also interacts with the metabolic regulator, FGF-21^11^. Serum levels of FGF-21 have been shown to increase in subjects with metabolic syndrome and atherosclerosis^12^. The pathophysiological conditions of these diseases have strong associations with smoking. Others and we previously reported the effects of smoking on subclinical atherosclerosis, visceral fat accumulation, and metabolic syndrome^13^,^14^,^15^. Therefore, we further evaluated the association between FGF-21 and smoking in the present study.

Cigarette smoking is known to stimulate the release of pro-inflammatory cytokines such as interleukin (IL)-6 and promote inflammation^16^,^17^. Recent studies have indicated that some growth factors trigger the release of pro-inflammatory cytokines and inflammation-related feedback loop^18^,^19^,^20^,^21^. In the present study, we also assessed the relationship between IL-6 and growth factors to evaluate the concern of inflammation in aging.

## Results

### Comparing the biochemical parameter among smokers and never-smokers

Characteristics of the study subjects are shown in Table 1. The mean age of the subjects was 46.1 ± 5.1 years and no significant difference was seen among smokers and never-smokers. Serum levels of FGF-21, soluble αKl (sαKl), and IL-6 were significantly higher in smokers than in never-smokers (P = 0.032, P = 0.048, and P = 0.011). Adiponectin (APN) levels were slightly lower in smokers than in never-smokers; however, the difference was not statistically significant (P = 0.20). The details of smoking habit in smokers are summarized in Table 2. We confirmed the correlation between smoking severity and serum levels of FGF-21, sαKl, and IL-6. Serum levels of IL-6 significantly correlated with smoking severity (r = 0.326, P = 0.046 and r = 0.301, P = 0.038), however FGF-21 and sαKl did not show any correlations (see Supplementary Table S1 online). Since serum levels of soluble αKl (sαKl) is reported to decrease with age^7^ and there were some age range (40–57 years) in the study subjects, we initially examined the correlation between age and sαKl in the subjects and confirmed that there were no correlation in both smokers and never-smokers (see Supplementary Table S2 online).

### Correlation between individual cytokines

Since FGF-21, sαKl, and IL-6 exhibited significant differences between smokers and never-smokers, we analyzed the correlations between each cytokine (n = 80). Serum levels of FGF-21 were correlated only with APN (r = −0.25, P = 0.013), and serum levels of sαKl were correlated only with IL-6 (r = 0.32, P < 0.001) (Table 3 and Supplementary Figure S1 online). It is well known that APN is a metabolic syndrome-related cytokine^22^, and that IL-6 is an inflammation-related cytokine^23^. These results suggest that FGF-21 is associated with metabolic syndrome and sαKl is associated with inflammation.

### FGF-21 and biochemical metabolic parameters

The correlations between serum levels of FGF-21 and biochemical metabolic parameters among smokers (n = 40) and never-smokers (n = 40) are summarized in Table 4. Serum levels of FGF-21 were significantly correlated with alanine transaminase (ALT) (r = 0.30, P = 0.008), γ guanosine-5′-triphosphate (GTP) (r = 0.39, P < 0.001), and total cholesterol (TC) (r = 0.34, P = 0.003), only in smokers. FGF-21 had been reported to associate with metabolic disorders^24^; however, as shown in Table 4, this correlation was only observed in smokers.

### Serum levels of α-klotho and inflammation-related stresses

**Figure 1:**
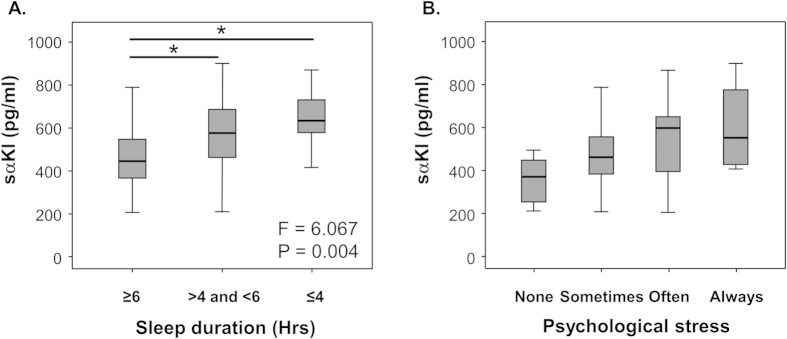
Relationship between serum levels of soluble alpha Klotho (sαKl) and environmental stresses. Relationships between serum levels of sαKl and (**A**) sleep duration time and (**B**) psychological stress. Sleep duration time was classified into three periods: ≥6 hours per day, >4 and <6 hours per day, and ≤4 hours per day. Psychological stress was classified into four stages: almost no stress (None), sometimes feel stress (Sometimes), often feel stress (Often), always feel stress (Always). Data are sample minimum, lower quartile, median, upper quartile, and sample maximum. n = 80, *P < 0.05 versus subjects whose sleep duration time were ≥6 hours per day.

As serum levels of sαKl correlate with inflammation-related cytokine IL-6, we analyzed the relationship between sαKl and other environmental stresses which relate with inflammation, such as sleep deprivation and severity of psychological stress^25^,^26^ (Fig. 1, n = 80). The serum levels of sαKl significantly increased in subjects with sleep deprivation (P = 0.004). In addition, the subjects who reported feeling considerable psychological stress, sαKl had a tendency to increase. Thus, not only smoking but also other inflammation-related environmental stresses, such as sleep deprivation and psychological stress, influenced the serum levels of sαKl.

### **α**-klotho and inflammation triggering growth factors

**Figure 2:**
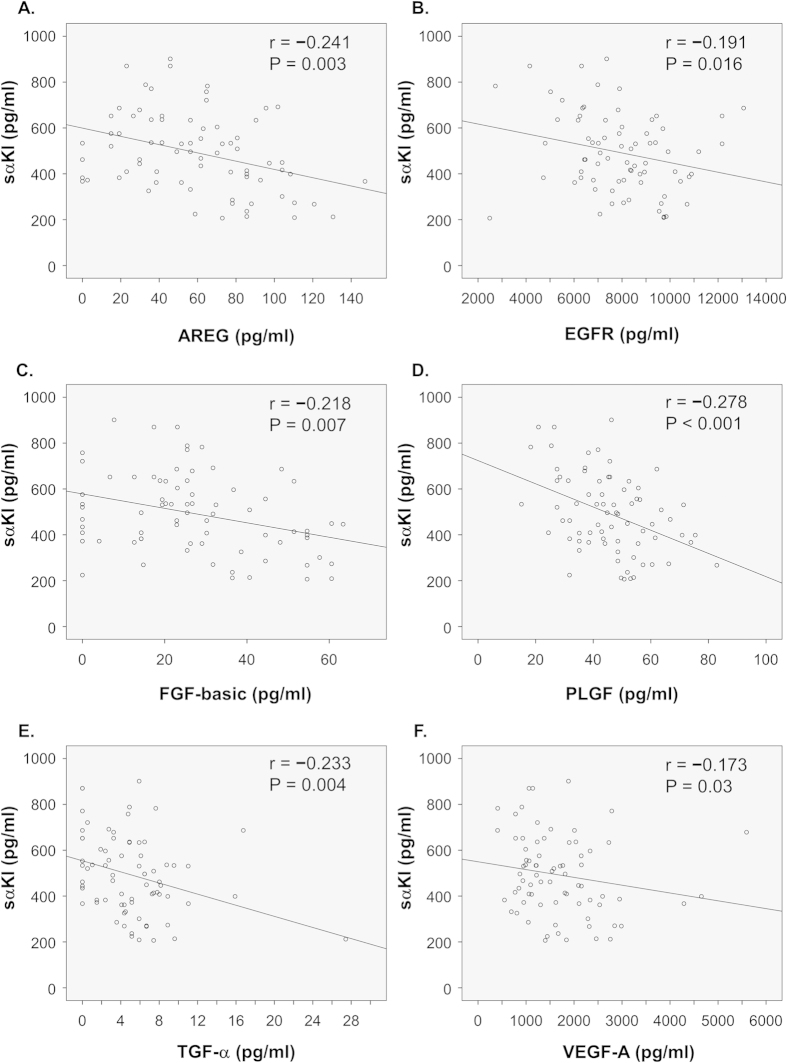
Correlations between serum levels of soluble alpha Klotho (sαKl) and growth factors. Correlations between serum levels of sαKl and growth factors. (**A**) amphiregulin (AREG), (**B**) epidermal growth factor receptor (EGFR), (**C**) fibroblast growth factor-basic (FGF-basic), (**D**) placental growth factor (PLGF), (**E**) transforming growth factor alpha (TGF-α), and (**F**) vascular endothelial growth factor (VEGF)-A. n = 80.

Figure 2 shows the correlations between serum levels of sαKl and various inflammation triggering growth factors among the subjects (n = 80). There was a significant negative correlation between sαKl and several growth factors: amphiregulin (AREG) (r = −0.241, P = 0.003) (A), epidermal growth factor receptor (EGFR) (r = −0.191, P = 0.016) (B), FGF-basic (r = −0.218, P = 0.007) (C), placental growth factor (PLGF) (r = −0.278, P < 0.001) (D), transforming growth factor alpha (TGF-α) (r = −0.233, P = 0.004) (E), and vascular endothelial growth factor (VEGF)-A (r = −0.173, P = 0.03) (F). These data suggest that anti-inflammatory effect of sαKl might be associated with diminished production of these growth factors.

### Entirely no correlation between α-klotho and IL-6 in smokers and aged men

**Figure 3:**
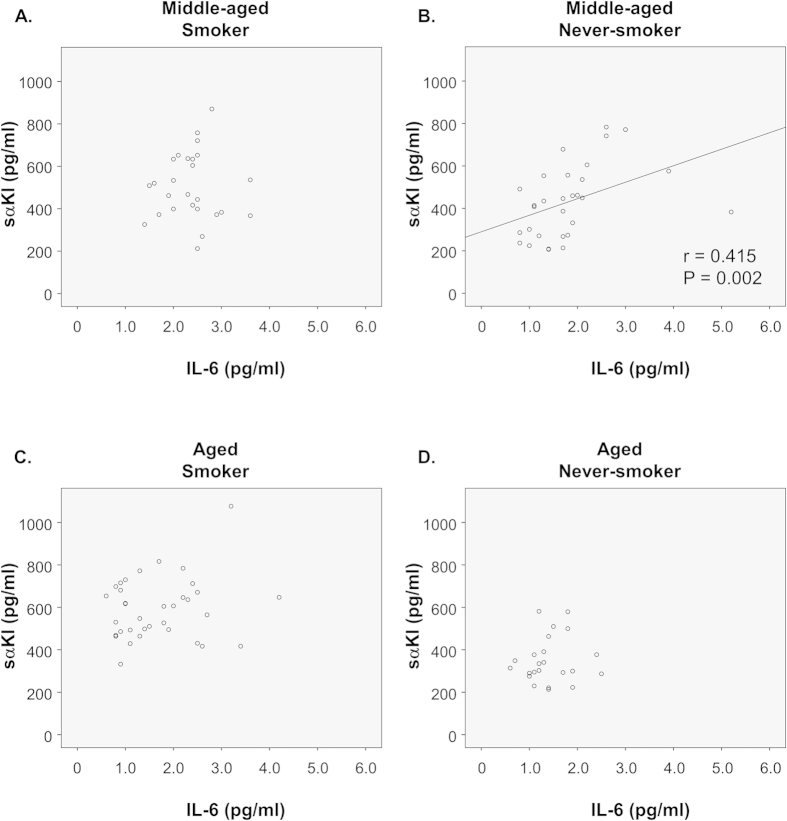
Correlations between serum levels of soluble alpha Klotho (sαKl) and interleukin (IL)-6 among smokers and never-smokers. Correlations between serum levels of sαKl and IL-6 in (**A**) middle-aged smoker (n = 40), (**B**) middle-aged never-smoker (n = 40), (**C**) aged smoker (n = 34), and (**D**) aged never-smoker (n = 26).

The correlations between serum levels of sαKl and IL-6 among smokers and never-smokers are shown in Fig. 3. There was a significant correlation between sαKl and IL-6 in never-smokers (B) (r = 0.415, P = 0.002); however, no significant correlation was found in smokers (A). In the preceding studies, we used middle-aged subjects (46.1 ± 5.1 years) for examination. In the following studies, we examined the serum levels of sαKl in aged subjects (60.3 ± 1.7 years) (see Supplementary Table S3, Supplementary Table S4, and Supplementary Figure S2 online). As shown in Supplementary Figure S2 online, serum levels of sαKl were significantly lower in aged never-smokers than in middle-aged never-smokers, confirming the previous report that serum levels of sαKl decrease along with aging^7^. However, aged smokers showed strongly and significantly increased serum levels of sαKl (P < 0.001). Unlike the clear correlation between sαKl and IL-6 in middle-aged never-smokers (Fig. 3 (B)), sαKl and IL-6 exhibited no correlation in aged subjects (Fig. 3 (C,D)). Furthermore, the correlation between sαKl and IL-6 seen with middle-aged smoker (Fig. 3 (A)) resembled to that seen with aged never-smokers (Fig. 3 (D)).

## Discussion

Aging is a progressive decline in physiological functions necessary for survival and fertility and is a multi-factorial phenomenon that is determined by various genetic and environmental factors^4^. Identification of the factors regulating aging is strikingly limited because aging is likely not mediated by a single gene or one specific mechanism. Smoking is considered as one of the environmental factors inducing aging. In appearance, compared to never-smokers, smokers tend to have more wrinkles, which is a characteristic of skin aging^27^. Other aging-related diseases, e.g., cancer, pulmonary disease, cardiovascular disease, osteoporosis, macular degeneration, and various brain pathologies are also frequently seen in smokers^3^. Moreover, previous reports have shown that compared to never-smokers, smokers have shorter life-spans^2^,^3^,^4^. While many hypotheses have been proposed to explain the aging process, the present study suggests that Klotho-related molecules might associate with the mechanism of aging accelerated by smoking habit.

The αKl gene-deficient mice show aging phenotypes such as atherosclerosis, osteoporosis, and pulmonary emphysema, which are also known as smoking-related diseases^5^,^6^. It has been reported that serum levels of sαKl change according to age and some disease states^7^,^28^; however, an association between sαKl levels and smoking has not been reported. To our knowledge, this is the first study to analyze the association between smoking and sαKl and the βKl-associated protein FGF-21.

We found that serum levels of FGF-21 were significantly higher in smokers. FGF-21 is a member of the FGF family, which is secreted from the liver and plays a role in glucose and lipid homeostasis^12^. FGF-21 binds the FGF receptors, which in turn interact with βKl, a homolog of αKl^11^. Serum levels of FGF-21 have been shown to increase in subjects with metabolic syndrome, non-alcoholic fatty liver, hyperlipidemia, hypertension, and atherosclerosis^12^,^24^. Although the precise function of FGF-21 is still unclear, this molecule is currently regarded as a useful biomarker of metabolic disorders. In the present study, FGF-21 was negatively correlated with APN, which is known to be decreased in metabolic syndrome^22^. However, FGF-21 was surprisingly correlated with liver function and TC only in smokers, and no significant correlation was observed in never-smokers. This result suggests that smoking stress might strengthen the relationship between FGF-21 and metabolic disorders. Although study subjects were healthy population which are not presenting any signs of metabolic disorder, serum levels of FGF-21 were upregulated by smoking. Therefore, serum levels of FGF-21 might be a predictor of progressing metabolic disorder especially in male smokers. Since there are no prior studies that address the influence of smoking on the correlation between FGF-21 and metabolic disorders, further studies are definitively needed to clarify these relationships.

We also found that the serum levels of the anti-aging molecule αKl were significantly higher in smokers than in never-smokers. This result presented a paradox; because smoking habit accelerates aging, we initially hypothesized that sαKl levels would decrease in smokers. It is well known that cigarette smoking and various stressful situations induce pro-inflammatory cytokine release and promote inflammation^17^,^25^,^26^. A previous study reported that one of the notable inflammatory markers IL-6 promotes aging phenotypes^29^, and in the current study, sαKl was positively correlated with IL-6 (Table 3). Since sαKl has been reported to act as an anti-inflammatory molecule^30^, this positive correlation suggests the possibility that the increase in serum levels of sαKl might be a compensatory response to smoking stress. Moreover, serum levels of sαKl were remarkably high in subjects with inflammation-related stress, not only in smokers but also in subjects with sleep deprivation and high psychological stress. It has been reported that sleep deprivation and psychological stress result in the elevation of inflammatory markers, including IL-6^25^,^26^. These results strengthen the hypothesis that an increase in sαKl is a compensatory response to stress and that sαKl functions as an anti-inflammatory molecule.

Some growth factors are reported to trigger the release of pro-inflammatory cytokines and inflammation-related feedback loop^18^,^19^,^20^. We analyzed the correlation between sαKl and growth factors and found negative correlations between sαKl and several growth factors. These data suggest that sαKl acts as a suppressor of the growth factors, which would explain the anti-inflammatory effects of sαKl. Therefore, we hypothesize that pro-inflammatory cytokine release induced by smoking is partially downregulated by sαKl. In this study, we focused on the anti-inflammatory effects of sαKl and confirmed the relation of sαKl and several inflammation-related growth factors. SαKl is also known to suppress the signal of transforming growth factor-β (TGF-β), which plays an important role in smoking-related pulmonary diseases^31^,^32^. Thus, it is quite possible that sαKl prevents smoking-related pulmonary diseases by inhibiting TGF-β signaling. To determine this possibility, the relation of sαKl and TGF-β should be evaluated in our further study.

However, there is a question still remained. Since serum levels of sαKl were higher in smokers than in never-smokers, the anti-inflammatory effects of sαKl should be obvious in smokers. But practically, many systemic inflammatory changes exist in the smokers. To resolve the question, we separately analyzed the correlations between serum levels of sαKl and IL-6 among the smokers and never-smokers. SαKl and IL-6 were positively correlated only in never-smokers in middle-aged, but not in smokers. This result indicates that smokers have high serum levels of sαKl and IL-6, but with no correlation. There remains the possibility that the relationship between sαKl and IL-6 is negated by smoking stress.

We examined the serum levels of sαKl and IL-6 in aged subjects. While the serum levels of sαKl were significantly lower in aged never-smokers than in middle-aged never-smokers, aged smokers showed a strong increase in their serum levels of sαKl. Moreover in aged subjects, even among never-smokers, did not show correlation between sαKl and IL-6, contrasting the positive correlation between sαKl and IL-6 seen among middle-aged never-smokers. Furthermore, middle-aged smokers showed aged pattern. It is intriguing to speculate that smoking habit accelerates aging by the disruption of the correlation between sαKl and IL-6.

There are some study limitations in this study. We selected only men and healthy subjects for the study. As we reported previously^13^, men and women show different response to smoking. However, women especially in middle-aged or older, have some problems due to the change in their endocrine system; secretion of sex hormones and menopause^33^. To exclude these problems, we used men subjects in the current study. As we aimed to evaluate the pure influence by smoking, we selected healthy subjects for the study. However, the smoker population might be biased to higher serum levels of sαKl and be protected from the smoking-related diseases. To reveal these problems, we needed to investigate women and disease population in the further study. Moreover, as smoking influences variously on multiple organs and precise mechanism by which sαKl acts in the inflammatory response is still unclear, there might be some other possible mechanisms of up-regulated serum levels of sαKl in smokers. We are willing to clarify these mechanisms in the further study. In the current study, we demonstrated an increase in serum levels of sαKl. However, very recent study reported that αKl expression in bronchial epithelial cells decreased in the lung of smokers and COPD patients^34^. Further study is necessary to reconcile this difference.

In conclusion, to our knowledge, this is the first study demonstrating that smoking simultaneously increases two Klotho-related molecules; sαKl, capable of affecting anti-inflammatory cytokine network and FGF-21, a possible indicator of progressing metabolic disorder in men.

## Methods

### Study subjects

The subjects were individuals who underwent health examinations in the Osaka University Health Care Center in 2010. Subjects were apparently healthy Japanese men (40 smokers and 40 never-smokers), 40–57 years of age, did not take any chronic or frequent medicine from at least one year before visiting the health examinations, and did not suffer acute illness within two weeks. These information was obtained via questionnaires and also reconfirmed in expert interview by trained nurses. For the aged group, age above 57 were additionally selected in the last part of the study (36 smokers and 24 never-smokers). The subjects were randomly selected, however to evaluate the precise influence by smoking habit, age and BMI were matched among smokers and never-smokers. The methods were carried out in accordance with the Declaration of Helsinki and the ethics guidelines for clinical research from the Ministry of Health, Labour and Welfare and the Ministry of Education, Culture, Sports, Science and Technology. All experimental protocols in this study were approved by the Ethics Committee of Health Care Center, Osaka University and written informed consent was obtained from all subjects prior to participation in the study.

### Physical and biochemical parameters

BMI and WC were measured as anthropometric measurements. WC at the umbilical level was measured in the late exhalation phase in standing position. VFA was estimated by impedance method as described previously^35^.

Serum was collected from subjects after overnight fasting and kept at ≤−20 °C until assay. The serum IL-6 concentration was measured with a chemiluminescent enzyme immunoassay (CLEIA) system (Fujirebio Inc., Tokyo, Japan). Serum levels of APN, sαKl, FGF-19, and FGF-21 were measured with a sandwich enzyme-linked immunoassay (ELISA) system according to the manufacturer’s instructions (Otsuka Pharmaceutical Co., Tokushima, Japan, Immuno-Biological Laboratories, Takasaki, Japan, R&D Systems Inc., Minneapolis, USA). To measure the serum concentrations of growth factors, detection kit was obtained commercially (WideScreen Human Cancer Panel 2, Merck, Tokyo, Japan). The growth factors included in this kit were as follows: AREG, betacellulin, epidermal growth factor, EGFR, epiregulin, FGF-basic, heparin-binding epidermal growth factor, platelet-derived growth factor-BB, PLGF, tenascin C, TGF-α, and VEGF-A. The data were followed by analysis with a multiplex analysis device (Bio-Rad Laboratories, Tokyo, Japan).

### Lifestyle assessments

Information on medical history, use of medicines and personal smoking habit, sleeping duration time and psychological stress were obtained via questionnaires. Each information was reconfirmed in expert interview by trained nurses. Sleeping duration time and psychological stress were semi-quantified as following scale. Sleeping duration time: 1 = ≥6 hours per day, 2 = >4 and <6 hours per day, 3 = ≤4 hours per day, psychological stress: 1 = almost no stress, 2 = sometimes feel stress, 3 = often feel stress, 4 = always feel stress.

### Statistical analysis

Data were analyzed using SPSS Statics 19 (IBM Corp., Armonk, NY, USA). Student’s t-test or Mann-Whitney U test was used to assess the difference between two groups. Kendall’s rank correlation coefficient was used to analyze variables. ANOVA followed by Tukey’s post hoc test was used to assess multi-group comparisons. Statistical significance was set at P < 0.05.

## Additional Information

**How to cite this article**: Nakanishi, K. *et al.* Klotho-related Molecules Upregulated by Smoking Habit in Apparently Healthy Men: A Cross-sectional Study. *Sci. Rep.* **5**, 14230; doi: 10.1038/srep14230 (2015).

## Supplementary Material

Supplementary Information

## Figures and Tables

**Table 1:** Characteristics of the study subject.

	All	Smoker	Never-smoker	P-value
	(n = 80)	(n = 40)	(n = 40)	
Age (years)	46.1 ± 5.1	46.1 ± 5.0	46.1 ± 5.2	0.98
BMI (kg/m^2^)	23.5 ± 2.8	23.9 ± 3.0	23.1 ± 2.7	0.26
WC (cm)	82.7 ± 7.3	83.3 ± 7.1	82.0 ± 7.5	0.43
VFA (cm^2^)	91.4 ± 34.9	94.7 ± 33.7	87.3 ± 36.5	0.44
SBP (mmHg)	122 ± 14	123 ± 15	121 ± 12	0.59
DBP (mmHg)	78 ± 10	79 ± 10	78 ± 10	0.61
Cr (mg/dl)	0.8 ± 0.1	0.8 ± 0.1	0.8 ± 0.1	0.37
UA (mg/dl)	6.1 ± 1.3	6.2 ± 1.2	6.0 ± 1.5	0.57
TC (mg/dl)	206 ± 33	209 ± 36	203 ± 30	0.39
TG (mg/dl)	114 ± 89	125 ± 96	104 ± 82	0.15
HDL-C (mg/dl)	58 ± 15	59 ± 17	57 ± 13	0.67
FPG (mg/dl)	90 ± 19	92 ± 26	88 ± 6	0.37
HbA1c (%)	5.1 ± 0.8	5.2 ± 1.0	4.9 ± 0.3	0.09
FGF-19 (pg/ml)	205 ± 157	183 ± 117	227 ± 188	0.67
FGF-21 (pg/ml)	239 ± 167	*283 ± **194**	**198** ± **126**	**0.032**
sαKl (pg/ml)	500 ± 170	*537 ± **163**	**462** ± **172**	**0.048**
IL-6 (pg/ml)	2.1 ± 0.8	*2.4 ± **0.5**	**1.8** ± **0.9**	**0.011**
APN (μg/ml)	7.2 ± 3.0	6.7 ± 2.7	7.9 ± 3.3	0.20

Data are expressed as mean ± SD. *P < 0.05 versus never-smokers.

BMI, body mass index; WC, waist circumference; SBP, systolic blood pressure; DBP, diastolic blood pressure; VFA, visceral fat area; UA, uric acid; TC, total cholesterol; TG, triglycerides; HDL-C, high-density lipoprotein-cholesterol; FPG, fasting plasma glucose; FGF-19, fibroblast growth factor-19; FGF-21, fibroblast growth factor-21; sαKl, soluble alpha-Klotho; IL-6, interleukin-6; APN, adiponectin.

**Table 2:** Details of smoking habit in smokers.

Smoker (n = 40)	n (%)
Cigarettes smoked per day	
10–20 cigarettes	29 (72.5)
21–30 cigarettes	8 (20)
31–40 cigarettes	3 (7.5)
Duration of smoking	
5–10 years	1 (2.5)
11–15 years	4 (10)
16–20 years	6 (15)
>20 years	29 (72.5)

**Table 3:** Correlations between fibroblast growth factor (FGF)-21, soluble alpha Klotho (sαKl), and other cytokines.

	FGF-21		sαKl	
	r	P-value	r	P-value
FGF-19	−0.05	0.60	−0.03	0.78
FGF-21	−	−	−0.06	0.46
sαKl	−0.06	0.46	−	−
IL-6	0.06	0.51	**0.32	**<0.001**
APN	*−0.25	**0.013**	0.12	0.23

n = 80, *P  <  0.05, **P < 0.001.

Abbreviations are as in Table 1.

**Table 4:** Correlations between fibroblast growth factor (FGF)-21 and biochemical parameters.

	Smoker (n = 40)		Never-smoker (n = 40)	
	r	P-value	r	P-value
AST	0.18	0.12	0.09	0.42
ALT	*0.30	**0.008**	0.10	0.39
γGTP	**0.39	**<0.001**	0.12	0.29
TC	*0.34	**0.003**	0.02	0.83
TG	0.22	0.06	0.04	0.73
HDL-C	−0.15	0.18	−0.04	0.70
FPG	0.13	0.26	0.20	0.08
HbA1c	0.08	0.49	−0.01	0.95

*P < 0.01, **P < 0.001.

Abbreviations are as in Table 1.
