# Supplementary material for: Klotho-related Molecules Upregulated by Smoking Habit in Apparently Healthy Men: A Cross-sectional Study
Source: Sci Rep. 2015 Sep 18;5:14230. doi: 10.1038/srep14230 (PMC4585559; doi:10.1038/srep14230)
Supplement: Supplementary Information [file srep14230-s1.pdf]

Supplementary Information

Klotho-related Molecules Upregulated by Smoking Habit in Apparently Healthy Men: A Cross-sectional Study

Kaori Nakanishi, Makoto Nishida, Masaya Harada, Tohru Ohama, Noritaka Kawada, Masaaki Murakami, Toshiki Moriyama, Keiko Yamauchi-Takihara.

Supplementary Table S1. Correlation between smoking severity and serum levels of fibroblast growth factor (FGF)-21, soluble alpha Klotho (sαKl), and interleukin (IL)-6

Smoker (n = 40)	FGF-21		sαKl		IL-6	
	r	P-value	r	P-value	r	P-value
Cigarettes smoked per day	−0.05	0.71	0.01	0.95	***0.326**	**0.046**
Brinkman index	0.08	0.49	−0.01	0.91	***0.301**	**0.038**

* P<0.05.

FGF-21, fibroblast growth factor-21; sαKl, soluble alpha-Klotho; IL-6, interleukin-6

We examined the correlation between smoking severity (using the number of cigarette smoked per day and Brinkman index) and serum levels of fibroblast growth factor (FGF)-21, soluble alpha Klotho (sαKl), and interleukin (IL)-6. Serum levels of IL-6 significantly correlated with smoking severity, however FGF-21 and sαKl did not show any correlations.

Supplementary Table S2. Correlation between age and serum levels of soluble alpha Klotho (sαKl) in middle-aged group

	All (n = 80)		Smoker (n =40)		Never-smoker (n =40)	
	r	P-value	r	P-value	r	P-value
age	−0.05	0.55	0.02	0.90	−0.09	0.45

To confirm the age influence on serum levels of soluble alpha Klotho (sαKl) in the middle-aged subjects (46.1 ± 5.1 years), we examined the correlation between age and serum levels of sαKl. There were no correlation between age and sαKl in the study subjects.

Supplementary Table S3. Characteristics of the subjects in aged group

	All (n = 60)	Smoker (n = 36)	Never-smoker (n = 24)	P-value
Age (years)	60.3 ± 1.7	60.1 ± 1.7	60.4 ± 1.8	0.56
BMI (kg/m^2)	23.5 ± 2.2	23.7 ± 2.3	23.2 ± 2.1	0.34
WC (cm)	83.3 ± 6.8	84.3 ± 6.9	81.7 ± 6.4	0.14
SBP (mmHg)	126 ± 14	126 ± 16	126 ± 10	0.98
DBP (mmHg)	81 ± 10	82 ± 11	80 ± 9	0.42
Cr (mg/dl)	0.8 ± 0.1	0.8 ± 0.1	0.8 ± 0.1	0.14
UA (mg/dl)	5.8 ± 1.1	5.9 ± 1.1	5.6 ± 1.2	0.34
TC (mg/dl)	208 ± 30	209 ± 30	208 ± 29	0.92
TG (mg/dl)	125 ± 57	***138 ± 64**	**105 ± 36**	**0.02**
HDL-C (mg/dl)	56 ± 14	55 ± 16	56 ± 11	0.82
FPG (mg/dl)	97 ± 18	96 ± 20	97 ± 14	0.88
HbA1c (%)	5.3 ± 0.5	5.2 ± 0.6	5.4 ± 0.3	0.07
sαKl (pg/ml)	497 ± 175	****590 ± 143**	**350 ± 107**	**<0.001**
IL-6 (pg/ml)	1.6 ± 0.7	1.7 ± 0.9	1.4 ± 0.5	0.117

Data are expressed as mean ± SD. * P<0.05, ** P<0.001 versus never-smokers.

BMI, body mass index; WC, waist circumference; SBP, systolic blood pressure; DBP, diastolic blood pressure; UA, uric acid; TC, total cholesterol; TG, triglycerides; HDL-C, high-density lipoprotein-cholesterol; FPG, fasting plasma glucose; sαKl, soluble alpha-Klotho; IL-6, interleukin-6

Serum levels of triglycerides (TG) and soluble alpha Klotho (sαKl) were significantly higher in smokers than in never-smokers (P = 0.02 and P < 0.001).

Supplementary Table S4. Details of smoking habit in aged smokers

Smoker (n = 36)	n (%)
Cigarettes smoked per day	
10 - 20 cigarettes	30 (83.3)
21 - 30 cigarettes	3 (8.3)
31 - 40 cigarettes	3 (8.3)
Duration of smoking	
11 - 15 years	1 (2.8)
16 - 20 years	4 (11.1)
> 20 years	31 (86.1)

The details of smoking habit in aged smokers are shown in Supplementary Table S4.

Supplementary Figure S1. Correlation between serum levels of fibroblast growth factor (FGF)-21 and interleukin (IL)-6 and adiponectin (APN)

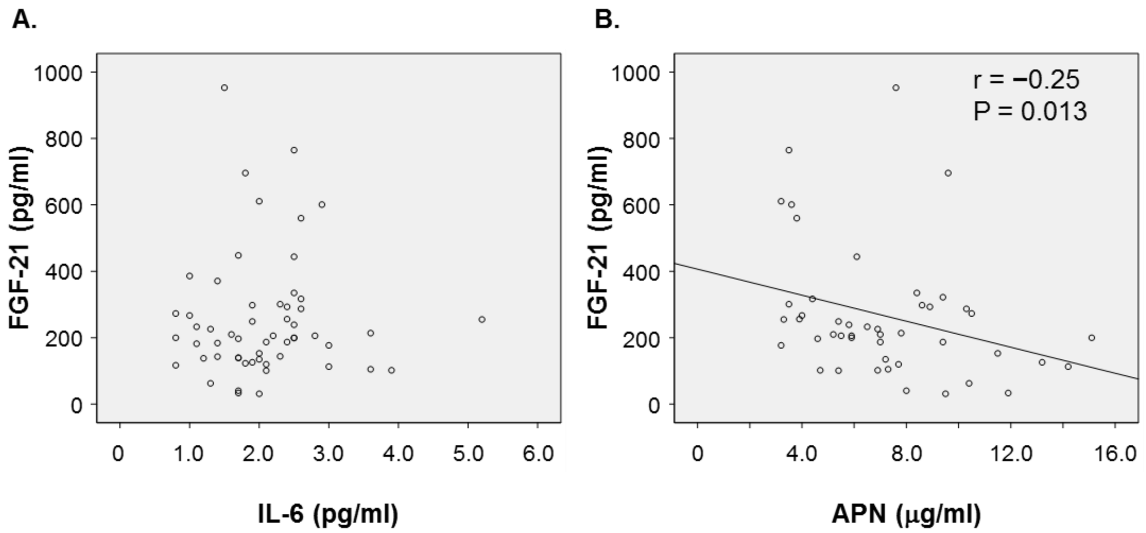

Correlations between serum levels of fibroblast growth factor (FGF)-21 and (A) interleukin (IL)-6 and (B) adiponectin (APN) (n = 80).

Serum levels of FGF-21 were correlated only with APN (r = −0.25, P = 0.013) and no significant correlation was found between FGF-21 and IL-6.

Supplementary Figure S2. Comparing the cytokines among middle-aged and aged subjects

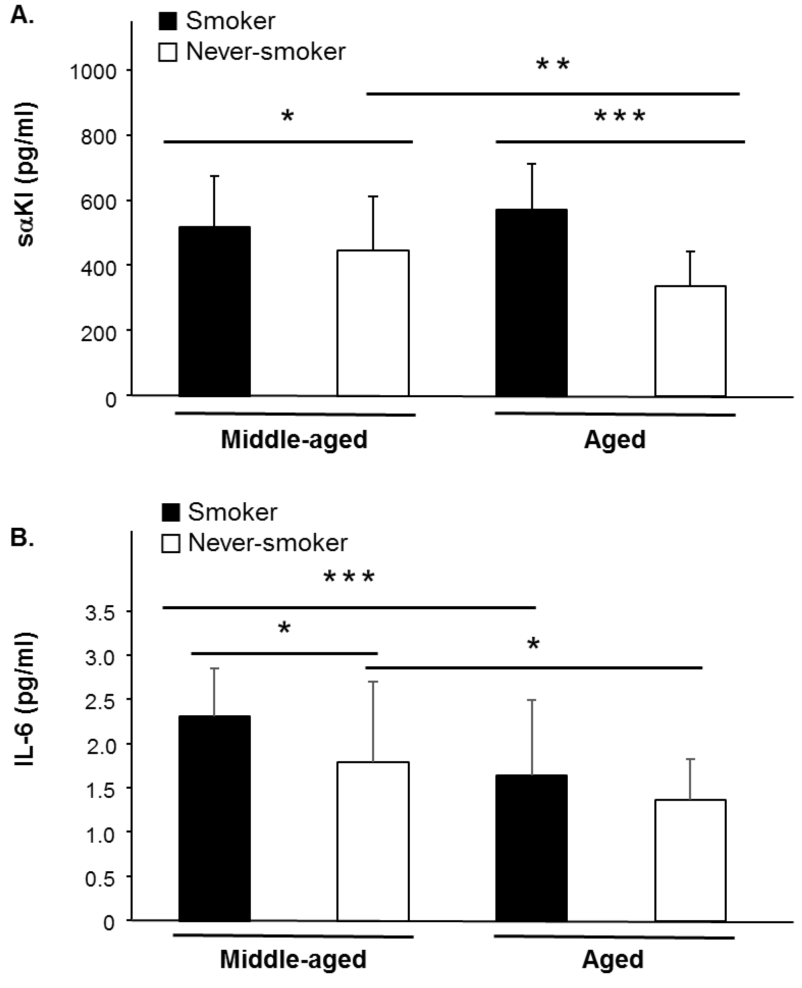

Comparing the serum levels of (A) soluble alpha Klotho (sαKl) and (B) interleukin (IL)-6 among middle-aged (46.1 ± 5.1 years, 40 smokers and 40 never-smokers) and aged (60.3 ± 1.7 years, 36 smokers and 24 never-smokers) subjects.

*P<0.05, **P<0.005, ***P<0.001.

In aged never-smokers, serum levels of sαKl and IL-6 were significantly lower to those of middle-aged never-smokers ($P = 0.002$, $P = 0.035$). Moreover, serum levels of IL-6 were significantly lower in aged smokers than in middle-aged smokers ($P <0.001$). However, aged smokers showed strongly and significantly increased serum levels of sαKl than aged never-smokers ($P < 0.001$).
